# In utero exposure to acetaminophen and ibuprofen leads to intergenerational accelerated reproductive aging in female mice

**DOI:** 10.1038/s42003-019-0552-x

**Published:** 2019-08-13

**Authors:** Moïra Rossitto, Margot Ollivier, Stéphanie Déjardin, Alain Pruvost, Christine Brun, Candice Marchive, Anvi Laetitia Nguyen, Aurélie Ghettas, Céline Keime, Bernard de Massy, Francis Poulat, Pascal Philibert, Brigitte Boizet-Bonhoure

**Affiliations:** 1 Development and Pathology of the Gonad, IGH, Centre National de la Recherche Scientifique, Université de Montpellier, Montpellier, France; 2 Service de Chirurgie et Urologie Pédiatrique, Hôpital Lapeyronie CHU Montpellier, Centre de Référence Maladies Rares Développement Génital, Montpellier, France; 3 Service de Pharmacologie et d’Immunoanalyse (SPI), plateforme SMArt-MS, CEA, INRA, Université Paris-Saclay, Gif sur Yvette, France; 4 Meiosis and Recombination, IGH, Centre National de la Recherche Scientifique, Université de Montpellier, Montpellier, France; 5 IGBMC, Centre National de la Recherche Scientifique, Université de Strasbourg/INSERM, Illkirch, France; 6 Département de Biochimie et Hormonologie, Hôpital Lapeyronie, CHU de Montpellier, Montpellier, France

**Keywords:** Oogenesis, Risk factors, Infertility

## Abstract

Nonsteroidal anti-inflammatory drugs (NSAIDs) and analgesic drugs, such as acetaminophen (APAP), are frequently taken during pregnancy, even in combination. However, they can favour genital malformations in newborn boys and reproductive disorders in adults. Conversely, the consequences on postnatal ovarian development and female reproductive health after in utero exposure are unknown. Here, we found that in mice, in utero exposure to therapeutic doses of the APAP-ibuprofen combination during sex determination led to delayed meiosis entry and progression in female F1 embryonic germ cells. Consequently, follicular activation was reduced in postnatal ovaries through the AKT/FOXO3 pathway, leading in F2 animals to subfertility, accelerated ovarian aging with abnormal corpus luteum persistence, due to decreased apoptosis and increased AKT-mediated luteal cell survival. Our study suggests that administration of these drugs during the critical period of sex determination could lead in humans to adverse effects that might be passed to the offspring.

## Abstract (web summary)

Moïra Rossitto et al. find that exposure of pregnant mice to acetaminophen and ibuprofen leads to delayed meiosis in the germline of female offspring and accelerated ovarian aging in F2 females. Their results suggest the use of these analgesics in pregnancy may adversely affect female reproductive health spanning multiple generations.

## Introduction

Nonsteroidal anti-inflammatory drugs (NSAIDs) such as ibuprofen (IBU) and analgesic drugs such as acetaminophen (APAP, or paracetamol) are among the most commonly prescribed drugs in the first trimester of pregnancy^1^. About 4% of pregnant women use APAP and NSAIDs in combination^2^. These are widely used to treat inflammation and pain^3^, and APAP and NSAIDs combinations are commonly prescribed to patients with fibromyalgia^4^ and acute pain^5^ to produce additive effects. However, these molecules can cross the placental barrier^6,7^. Epidemiological studies and experimental in vivo/ex vivo investigations in rodents, adult men^8^ and using human testicular explants^3,9^ have established a possible link between prenatal or adult exposure to these drugs and increased effects on the male reproductive tract formation and physiology, thus identifying these molecules as putative endocrine disruptors^3,8–10^. Also, early developmental exposure of mouse testes to the APAP–IBU combination affects adult sperm parameters in the exposed males and in their offspring^11^.

Perinatal exposure of female rodents to high doses of APAP reduces birth weight without affecting mouse fertility^12^ and also decreases the primordial follicle pool in pubertal rats, leading to early reproductive senescence in adults^13^. In utero exposure to APAP or indomethacin has been associated with a reduced number of primordial germ cells^14^ and delayed meiotic entry^15^ in fetal mouse and rat ovaries, respectively, leading to a decreased number of secondary follicles in adult mouse ovaries^14^ and reduced fertility in F1 and F2 rat females^15^. In ex vivo cultures of human embryonic ovaries exposed to APAP or IBU, the germ cell number is reduced due to alterations in their proliferation and apoptosis^16,17^. These studies suggest that the time windows corresponding to gonadal sex determination and meiosis entry are particularly sensitive to the effects of these molecules. However, the molecular bases of their effects, particularly when such drugs are used in combination, on the female reproductive health remain unknown^18,19^.

In mouse female embryonic gonads, sex determination starts with the specific expression of key ovarian somatic genes (e.g., *Rspo1*, *Wnt4* and *Ctnnb1*) at 11.5–12.0 days post coitum (dpc) that promote oogonial proliferation and survival, and germ cell meiosis initiation^20,21^. *FoxL2* is expressed at around 12.0 dpc, and plays an essential role in the maintenance of the granulosa cell ovarian phenotype in developing mouse ovaries^21,22^. At 12.5 dpc, female germ cells enter the meiosis I prophase^23^ and reach diplotene by 18.5 dpc. At that time, germ cell cysts, which are formed by interconnected germ cells, undergo breakdown until birth, and concomitantly, many germ cells are lost through apoptosis until day 4 post partum (dpp)^24,25^. At birth, single oocytes arrested in the meiotic diplotene/dictyate stage are rapidly surrounded by flat pre-granulosa cells to form primordial follicles (PFs)^26^. Most PFs remain quiescent, and only few will mature into primary follicles through the coordinated action of various somatic and oocyte factors^26,27^, such as Kit ligand that activates the PI3K/AKT/FOXO3 cascade^28^, a major signalling pathway involved in the regulation of follicle activation and survival^29^. Upon follicle maturation and ovulation, follicular cells differentiate to form the corpus luteum (CL), an endocrine organ that produces progesterone to support pregnancy^30^ and that regresses in the absence of pregnancy^31,32^.

The female reproductive lifespan is largely determined by the PF pool size that is established early in life^33,34^. Dysregulation of the signalling cascades that regulate cell proliferation and cell death and that determine whether a follicle will continue to develop or undergo atresia can disturb the balance between PF dormancy and activation^35^. Particularly, environmental factors acting during the prenatal or adult life seem to be major determinants of the ovarian reserve^19^ and thus, could contribute to the onset of premature ovarian insufficiency (POI)^35,36^. In this study, we evaluated whether and how in utero exposure to APAP and IBU affects the female reproductive system development by administering human-relevant doses of these drugs, adjusted for mice, alone or in combination to pregnant mice between 10.5 and 13.5 dpc, the period of sex determination. Upon exposure to the APAP + IBU combination, germ cell proliferation and meiosis entry/progression were affected in embryonic ovaries, and PF formation and activation were abnormal in postnatal ovaries. Consequently, the ovarian reserve decreased progressively in exposed (F1) animals and their offspring (F2), leading to subfertility in 6-month-old F2 animals that showed accelerated ovarian aging and abnormal CL persistence.

## Results

### Increased germ cell proliferation in F1 APAP + IBU embryonic ovaries

**Fig. 1:**
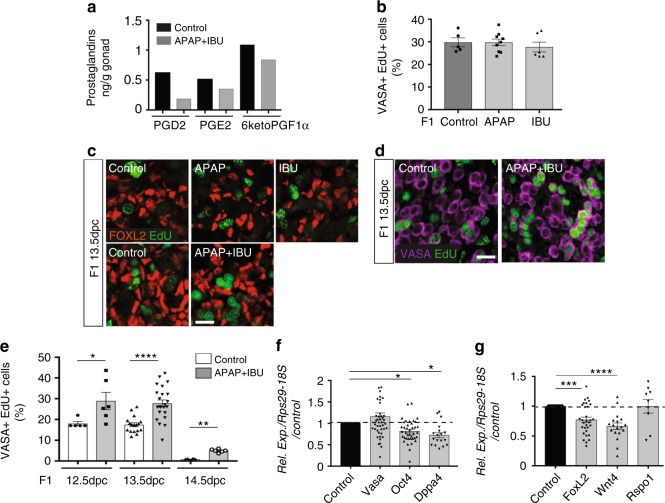
In utero exposure to APAP and NSAIDs increases female germ cell proliferation. **a** Dosage of PGD_2_, PGE_2_ and 6-keto-PGF_1α_ (PGI_2_) by mass spectrometry in F1 13.5 dpc control and APAP + IBU-exposed ovaries (*n* = 1 pool of 200 and 220 gonads from 15 control and 15 treated pregnant females, respectively). **b** Quantification of VASA^+^ EdU^+^ cells in F1 control and APAP or IBU-exposed ovaries; data are the percentage of proliferating EdU^+^ cells among all VASA^+^ cells (*n* = 20–25 gonads from *n* = 12 litters). **c**, **d** Representative immunofluorescence microscopy images of F1 13.5 dpc ovaries from embryos in utero exposed to ethanol (Control), APAP, IBU or APAP + IBU between 10.5 and 13.5 dpc and pulsed with EdU at 13.5 dpc; FOXL2 (granulosa cell marker) in red (**c**), VASA (germ cell marker) (**d**) in purple and EdU in green (scale bars, 20 μm). **e** Percentage of proliferating EdU^+^ cells among all VASA^+^ cells in F1 control and exposed ovaries at 12.5, 13.5 and 14.5 dpc (EdU pulse at 12.5, 13.5 and 14.5 dpc, respectively) (*n* = 16–20 gonads from *n* = 8 litters). **f**, **g** Expression analysis of *Vasa, Oct4, Dppa4* (**f**)*, Foxl2, Wnt4* and *Rspo1* (**g**) in F1 exposed ovaries, normalised to *Rps29* and *18S* expression and presented as percentage of the expression in control 13.5 dpc ovaries (set to 1). Values are the means ± SEMs; *P < 0.05, **P < 0.01, ***P < 0.005, ****P < 0.001 (**e**–**g**)

To evaluate the effect of APAP and/or IBU exposure on early ovarian development, we administered APAP (30 mg/kg/day) or IBU (15 mg/kg/day) as single drugs (two gavages per day for each drug) or in combination (four gavages per day every 3 h: two for each drug) to pregnant mice between 10.5 and 13.5 dpc (i.e., the sex determination period). Controls received diluent alone. We previously reported that at 30 min after oral administration, APAP and IBU serum levels in pregnant mice^11^ are similar to those reported in a xenograft mouse model^37^ and in the umbilical cord of human fetuses^16^. Furthermore, we detected these drugs also in the embryos’ liver^11^, confirming that they can cross the placental barrier^6,7^. Prostaglandin (PG) quantification in 13.5 dpc ovaries showed that production of PGD_2_, PGE_2_ and 6-keto-PGF_1α_, an inactive hydration product of PGI_2_^38^, was decreased by 70%, 32 and 23%, respectively, in 13.5 dpc APAP + IBU-exposed ovaries compared with control (Fig. 1a). We could not detect 15-desoxy-PGJ_2_ (an active PGD_2_ metabolite), PGF_2α_ and TXA2 in control and exposed ovaries.

Co-immunofluorescence analysis of 13.5 dpc female gonad sections using antibodies against FOXL2 (granulosa cell marker) or VASA/MVH (germ cell marker) concomitantly with EdU labelling (S-phase cells) did not highlight any significant difference in the proliferation rate of F1 germ cells (VASA^+^-EdU^+^) and granulosa cells (FOXL2^+^-EdU^+^) in 13.5 dpc gonads exposed to one single drug and control (Fig. 1b, c). Conversely, the percentage of the S-phase germ cells (VASA^+^-EdU^+^) (Fig. 1d, e), but not of granulosa cells (FOXL2^+^-EdU^+^) (Fig. 1c), was significantly higher in F1 gonads exposed to the APAP + IBU combination. Germ cell proliferation was higher also in 12.5 and 14.5 dpc APAP + IBU-exposed ovaries (Fig. 1e), suggesting a delay in germ cell differentiation (i.e., meiosis entry), which normally initiates at 12.5 dpc. On the other hand, expression of *Ddx4 (Vasa/Mvh)* was not modified, whereas that of the *Pou5f1* (*Oct4)* and *Dppa4* pluripotent genes was significantly decreased in 13.5 dpc APAP + IBU ovaries compared with controls (Fig. 1f), suggesting that pluripotency downregulation might be anticipated. The expression level of the *Wnt4* and *FoxL2* ovarian genes also was significantly affected, but not that of *Rspo1* (Fig. 1g), suggesting that differentiation of the ovarian somatic cell lineage could also be affected.

Transcriptome analysis of 13.5 dpc female gonads showed that compared with controls, 2215 genes were downregulated (<0.75-fold) and 2246 were upregulated (>1.33-fold) in APAP + IBU ovaries (*P*-value < 5 × 10^–5^) (Supplementary Data 1) (data accessible through the GEO series accession number GSE122547. Gene ontology analysis using the ToppGene program^39^ indicated that APAP + IBU exposure influenced many biological pathways involved in cellular metabolism (downregulated genes) and meiotic recombination and meiotic cell cycle (upregulated genes) (Supplementary Table 1). Among the 104 genes specific to meiotic prophase that have been identified by Soh et al.^40^, two [stimulated by retinoic acid gene 8 *(Stra8*; 0.76-fold) involved in meiosis initiation; and meiotic recombination protein Rec8 homologue *(Rec8*; 0.65-fold)] were downregulated and 47 were upregulated in APAP + IBU-exposed ovaries (Supplementary Table 2). Several of the upregulated genes were involved in meiosis progression^40^, such as deleted in azoospermia-like *(Dazl*; 1.5-fold), *Sycp1* (2.82-fold), *Sycp2* (2.45-fold) and *Sycp3* (1.78-fold) (three genes encoding synaptonemal complex proteins), double-strand-break repair rad21-like protein 1 (*Rad21I*; 3.12-fold), spermatogenesis-associated protein 22 *(Spata22*; 2.66-fold), meiotic protein covalently bound to DSB *(Spo11*-1.77-fold), Horma domain-containing proteins 1 and 2 *(Hormad1*, 1.74-fold, and *Hormad2*, 1.45-fold), meiosis specific with coiled–coil domain (*Meioc*, previously identified as *Gm1564*, 1.89-fold), DNA repair protein Rad51 associated protein *(Rad51ap2*; 3.15- folds) and meiotic recombination protein Dmc1/Lim15 homologue *(Dmc1*; 1.77-fold) (Supplementary Table 2).

**Fig. 2:**
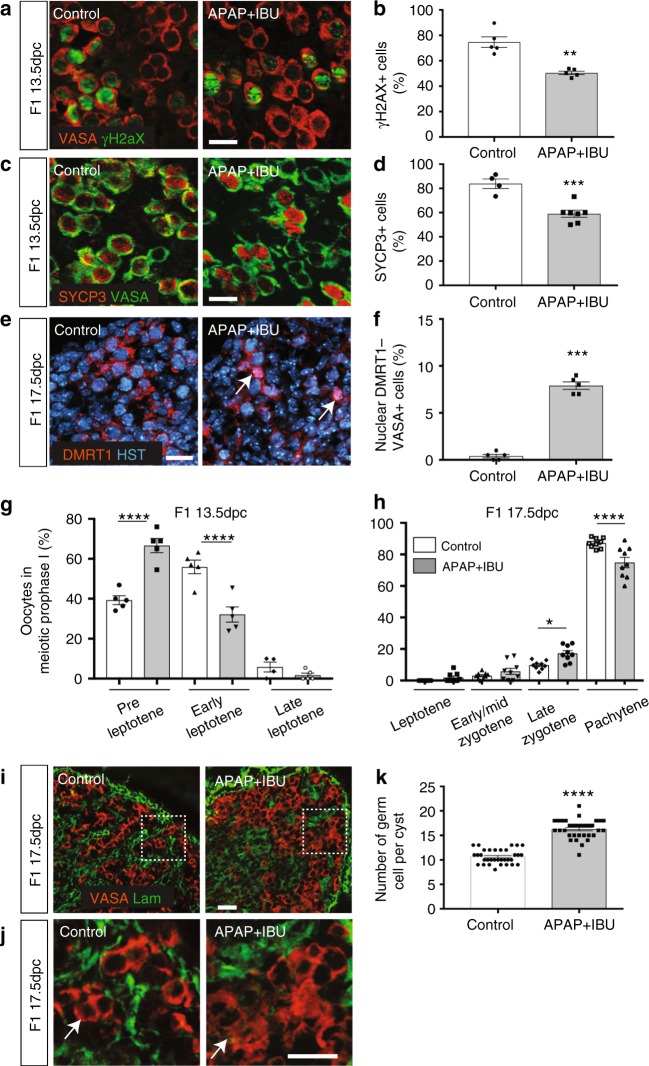
In utero exposure to APAP + IBU impairs meiosis progression. **a**, **c**, **e**, **i** Representative immunofluorescence images of γH2AX (green, **a**), SYCP3 (red, **c**), DMRT1 (red, **e**) and laminin (green, **i**) together with VASA (**a**, **c**, **i**) or Hoechst dye (HST, blue) (**e**) in tissue sections from F1 13.5 dpc (**a**, **c**) and F1 17.5 dpc (**e**, **i**, **j**) control and APAP + IBU ovaries. Enlarged panels (**j**) delineated from panel (**i**) (white dashed squares) show germ cell cysts. Scale bars = 20 μm. White arrows indicated germ cells with nuclear DMRT1 expression (**e**) and within a cyst (**j**). **b**, **d** Quantification of γH2AX^+^ (**b**) and SYCP3^+^ (**d***)* cells among all VASA^+^ germ cells in control and in utero exposed ovaries; data are the percentage relative to all VASA^+^ cells. **f**, **k** Quantification of VASA^+^ germ cells with nuclear DMRT1 expression (**f**) and in cysts (**k**) in F1 17.5dpc ovaries. **b**, **d**, **f** Values are the mean ± SEMs of *n* = 16–24 gonads from *n* = 8 litters, **P < 0.01, ***P < 0.005; **k** Values are the mean ± SEMs of *n* = 10 (control) and *n* = 12 (APAP + IBU) gonads from *n* = 3 litters, ****P < 0.001. **g**, **h** Quantification of oocytes of meiotic prophase I on nuclear spreads of F1 13.5 dpc (**g**) and F1 17.5 dpc (**h**) ovaries. The data are presented as the percentage of oocytes at the preleptotene, early leptotene and late leptotene stages per total number of analysed oocytes (*n* = 85 to 105 for 13.5 dpc gonads) (**g**) and of oocytes at the leptotene, early/mid zygotene, late zygotene and pachytene stages per total number of analysed oocytes (*n* = 110 to 155 for 17.5dpc gonads) (**h**). *P < 0.05, ****P < 0.001

Therefore, we analysed the meiosis entry in F1 13.5 dpc-exposed gonads. Immunofluorescence analysis with antibodies against VASA and γH2AX (a marker of DNA double-strand breaks during early meiotic recombination)^41^ (Fig. 2a, b) or SYCP3 (a leptotene oocyte marker)^42^ (Fig. 2c, d), showed that the percentage of γH2AX^+^ and of SYCP3^+^ among VASA^+^ cells, was significantly decreased in 13.5 dpc APAP + IBU-exposed ovaries compared with controls, suggesting a delay in meiosis entry in exposed ovaries. Following sex determination, the meiosis regulator DMRT1 is expressed only in female germ cells^43^, where it is relocated from the nucleus to the cytoplasmic compartment after meiosis initiation (after 14.5 dpc in mice)^44^. In F1 17.5 dpc APAP + IBU-exposed ovaries, DMRT1 was localised in the nucleus in 9% of VASA^+^ germ cells whereas in controls, it was expressed only in the cytoplasm, indicating that some exposed germ cells were still initiating meiosis at this stage (Fig. 2e, f). We next assessed the progression of oocyte meiotic prophase using surface spreads from 13.5 dpc and 17.5 dpc ovaries and staining with anti-γH2AX, SYCP3 and SYCP1 antibodies to analyse DNA double-strand break and synaptonemal complex (SC) formation (Supplementary Fig. 1a). At 13.5 dpc, in the exposed APAP + IBU compared with the control group, the percentage of oocytes at the preleptotene stage was increased (66.6% vs. 39.3%; *P*-value < 0.0001), whereas the percentage of oocytes at the leptotene stage was decreased (32.1% vs. 55.9%; *P*-value < 0.0001) (Fig. 2g). As the preleptotene stage is the stage of meiotic initiation, this result was compatible with a defect in entry into meiosis. At 17.5 dpc, the percentages of oocytes at early/mid zygotene, late zygotene and pachytene stages in the exposed vs. the control group were 3% vs. 5.85% (*P*-value = 0.92), 9.8% vs. 17.2% (*P*-value = 0.035) and 87.1% vs. 74.8% (*P*-value = 0.0001), respectively (Fig. 2h). These results indicate that APAP + IBU exposure induced a delay in meiotic entry and in progression during prophase I of oocytes. The slight increase of the late zygotene stage could be due to an extended zygotene stage upon treatment. Specifically, meiosis initiation is delayed, whereas expression of genes involved in meiosis progression was increased, suggesting a misregulation of the normal meiotic differentiation programme. The meiosis delay and the increased cell proliferation observed in F1 exposed germ cells at 13.5 dpc could be responsible for the higher number of germ cells in cysts of exposed ovaries at 17.5 dpc compared with controls (mean = 16.2 vs. 10.6 germ cells per section of exposed and control gonads, *P* < 0.0001) (Fig. 2i–k).

### Primordial follicle formation is increased in postnatal ovaries

**Fig. 3:**
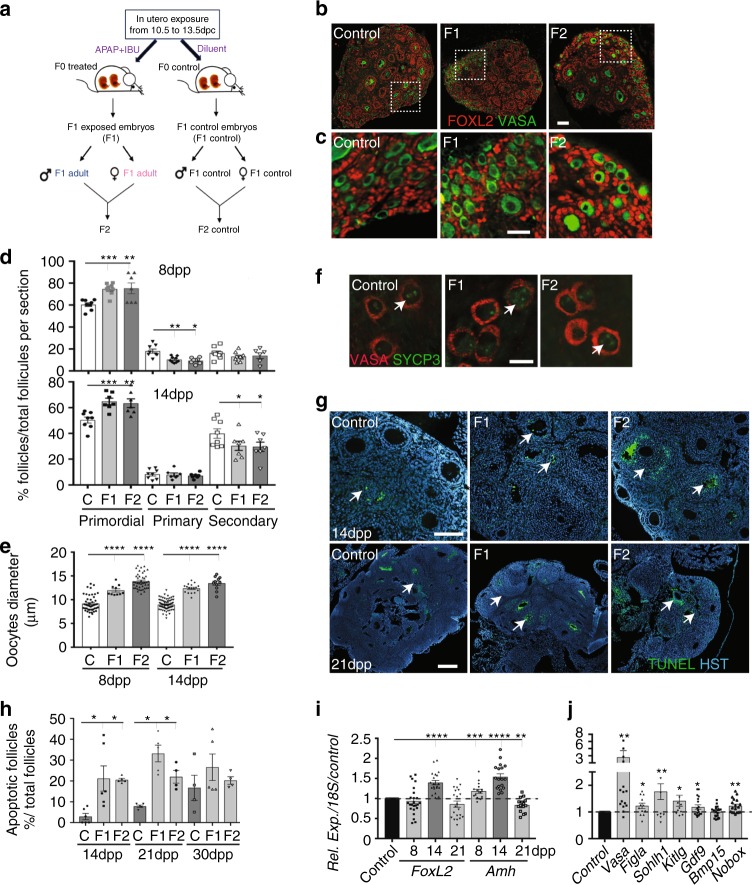
Primordial follicle formation and maturation are impaired in postnatal F1 and F2 ovaries. **a** Schematic representation of the experimental design to obtain F1 and F2 animals after in utero exposure to APAP + IBU. **b**, **c** Representative immunofluorescence images of 8 dpp control, F1 and F2 ovaries stained for FOXL2 (red) and VASA (green) (scale bars = 50 μm). Enlarged panels (**c**) highlight details of the ovarian cortex from panels in **b**. **d** Primordial, primary and secondary follicles per section of 8 dpp and 14 dpp control (C), F1 and F2 ovaries. The results are the percentage of primordial, primary and secondary follicles among all follicles (*n* = 16–20 different ovaries per data point). **e** Oocyte diameter is increased in 8 dpp and 14 dpp F1 and F2 ovaries (*n* = 20 ovaries and 200 oocytes per data point); ***P < 0.005, ****P < 0.001. **f** SYCP3 (green) and VASA (red) signal in 8 dpp control, F1 and F2 ovary sections. White arrows indicated germ cells in the diplotene/dictyate stage. Scale bar = 10 μm. **g** Representative immunofluorescence images of TUNEL assays in 14 and 21 dpp ovaries. White arrows indicate apoptotic follicles. Scale bars = 60 μm. **h** Percentage of atretic follicles relative to all follicles in 14 dpp, 21 dpp and 30 dpp control, F1 and F2 ovaries; *P < 0.05. **i**, **j** Expression levels of *FoxL2* and *Amh* in 8, 14 and 21 dpp control and F1 ovaries (**i**) and of germ cell/somatic genes in 8 dpp control and F1 ovaries (**j**). The data are the ratio between F1 and control expression (**h**). *P < 0.05, **P < 0.01, ***P < 0.005, ****P < 0.001

Then, we determined whether these germ cell defects affected the oocyte and follicle development also after birth. As we previously observed intergenerational effects of APAP + IBU exposure on mouse testis maturation and function^11^, we analysed F1 postnatal ovaries of animals that were in utero exposed and also of their offspring (F2) (both F1 parents exposed) relatively to their respective controls, F1 control and F2 control (Fig. 3a).

We analysed the first wave of folliculogenesis in 8, 14, 21 and 30 dpp F1 and F2 ovaries. As the phenotypes of F1 control and F2 control animals/ovaries were similar, data from these two groups were globally referred to as controls. Histological analysis showed that the morphology of postnatal ovaries (PFs and primary follicles in 8 and 14 dpp ovaries, secondary follicles in 21 and 30 dpp ovaries) was similar in controls, F1 and F2 animals (Supplementary Fig. 2a). Expression of FOXL2^45^ and AMH^46^, two granulosa cell markers, and of COUPTFII, a theca and interstitial cell marker^47^, was comparable in primary and secondary follicles of 8 and 14 dpp F1 and F2 and control ovaries (Fig. 3b, c; Supplementary Fig. 2b). Conversely, more PFs were present in 8 and 14 dpp F1 and F2 ovaries than in controls (Fig. 3b–d), whereas the opposite was true for primary follicles at 8 dpp and for secondary follicles at 14 dpp (Fig. 3d). Also, the oocyte diameter in PFs was significantly higher in F1 and F2 ovaries than in controls (Fig. 3e), suggesting increased oocyte activation. Meiosis I prophase was correctly achieved because SYCP3 staining^48^ confirmed that 8 dpp oocytes were blocked at the diplotene/dictyate stage (Fig. 3f). However, the apoptosis rate (TUNEL staining) within primary and secondary follicles was significantly higher in 14 and 21 dpp (but not in 8 and 30 dpp) F1 and F2 ovaries compared with controls (Fig. 3g, h). Moreover, *FoxL2* and *Amh* expression was significantly increased in 14 dpp (*FoxL2*) and in 8 and 14 dpp (*Amh*) F1 ovaries compared with controls, whereas *Amh* expression was decreased in 21dpp F1 ovaries (Fig. 3i). Moreover, *Vasa* was upregulated in 8 dpp-exposed ovaries, in agreement with their higher number of oocytes at this stage (Fig. 3j). Accordingly, the expression of folliculogenesis-specific basic helix–loop–helix (*Figla*) and spermatogenesis and oogenesis bHLH transcription factor *1* (*Sohlh1)*, two genes implicated in PF formation^49,50^, was significantly increased in F1 ovaries (8 dpp) compared with controls (Fig. 3j). Expression of the genes encoding KIT ligand (*Kitlg)*, oogenesis homeobox protein NOBOX (*Nobox*) and growth differentiation factor 9 (*Gdf9*), which are critical for the formation of primary follicles^27^, was slightly but significantly increased in F1 ovaries compared with controls (8dpp) (Fig. 3j). These results suggest an increase of the PF pool size and of oocyte activation in 8 dpp F1 and F2 ovaries. Concomitantly, the increased apoptosis rate and *Amh* expression, which is known to inhibit follicle activation^51^, might explain the lower number of primary and secondary follicles in 8 and 14 dpp F1 and F2 ovaries.

### Altered activation of the AKT/FOXO3 pathway in F1 postnatal ovaries

**Fig. 4:**
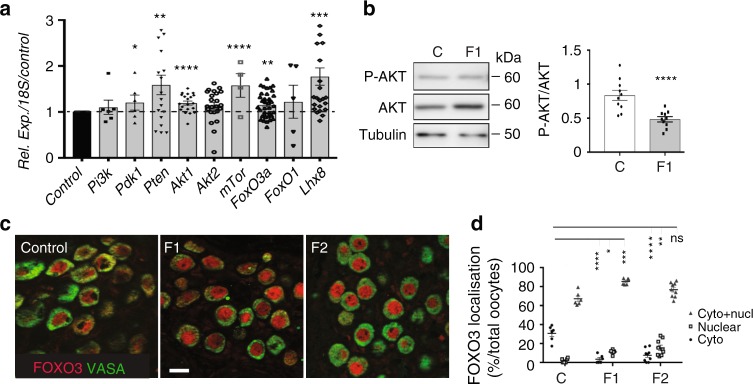
Inhibition of the AKT-FOXO3 pathway in oocytes after in utero exposure to APAP + IBU. **a** Relative gene expression of PI3K/AKT pathway components in 8 dpp control and F1 ovaries. **b** Representative immunoblots of the total AKT, phosphorylated AKT (P-AKT) and tubulin expression in 8 dpp control and F1 protein extracts (*n* = 2 pools of two ovaries) and quantification of the P-AKT/AKT ratio; ****P < 0.001. **c** Representative immunofluorescence images of 8 dpp control, F1 and F2 ovaries stained for FOXO3 (red) and VASA (green) (scale bar = 15 μm). **d** Quantification of nuclear, cytoplasmic and nuclear/cytoplasmic FOXO3 expression in oocytes of control, F1 and F2 ovaries (8 dpp) (*n* = 260, 330 and 500 oocytes, respectively). The data are expressed as the percentage of all oocytes and as the mean ± SEMs; *P < 0.05, **P < 0.01, ***P < 0.005, ****P < 0.001

We then analysed the expression of the PI3K/AKT/FOXO3 pathway that is critical for PF pool maintenance^29,52^. Expression of the genes encoding phosphatase and tensin homologue deleted on chromosome 10 (*Pten)*^53^, mammalian target of rapamycin complex 1 (*mTorC1)*^54^ and LIM homeobox protein (*Lhx8)*^55^, three major repressors of oocyte activation, was significantly increased in 8 dpp F1 ovaries compared with controls (Fig. 4a). Expression of the genes encoding phosphatidylinositol-dependent kinase 1 (*Pdk1)* and Akt kinases, that activate this pathway, as well as of forkhead transcription factor *FoxO3*, an effector of this pathway and a specific AKT substrate, was only slightly increased in 8 dpp F1 ovaries (Fig. 4a). AKT phosphorylation analysis by western blotting in 8 dpp control and F1 ovaries showed a significant decrease by 35% of the phosphorylated AKT/total AKT ratio in F1 ovaries compared with controls (mean of 0.161 vs. 0.103), indicating reduced AKT activation (Fig. 4b; Supplementary Fig. 3). Consequently, FOXO3 nuclear expression was significantly increased in 8 dpp F1 compared with control oocytes (11.2% of F1 oocytes and 2% of controls), whereas FOXO3 cytoplasmic expression was significantly reduced (3.5% of F1 oocytes and 31% of controls) (Fig. 4c, d). These defects were observed also in 8 dpp F2 oocytes compared with controls (nuclear FOXO3 expression: 15.5% of F2 and 2% of control oocytes; cytoplasmic FOXO3 expression: 7.7% of F2 and 31% of control oocytes) (Fig. 4c, d). Altogether, these results suggest that the transition from PF to primary follicles might be hindered in the ovaries of APAP + IBU-exposed animals through inactivation of the AKT/FOXO3 pathway and consequently, inhibition of FOXO3 nucleocytoplasmic translocation. Moreover, these defects are transmitted to the offspring.

### *S*ubfertility in F2 females that show accelerated ovarian aging

**Fig. 5:**
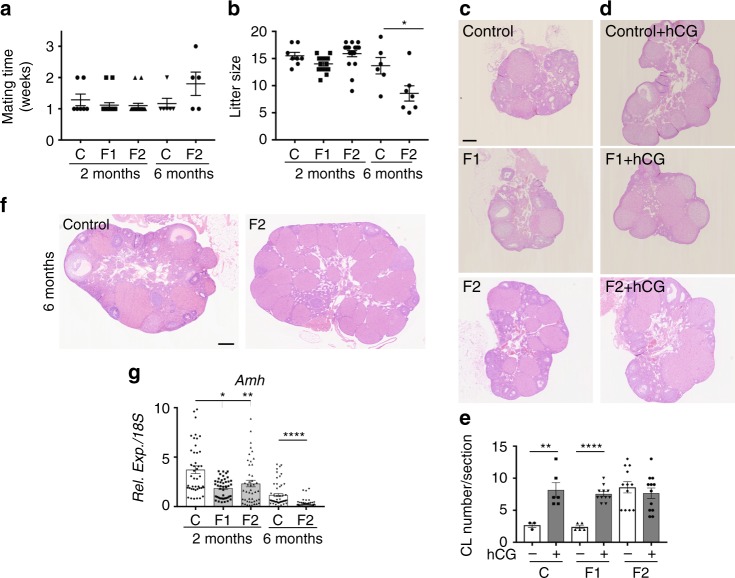
F2 females show accelerated ovarian aging and are subfertile. **a**, **b** Fertility of 2-month- and 6-month-old females was determined in terms of time to mating (**a**) and litter size (**b**) (*n* = 6–19 for each data point) *P < 0.05. **c**, **d**, **f** Histological analysis of 2-month-old ovaries stimulated (**d**) or not (**c**) by hCG and of 6-month-old ovaries (**f**). All sections were stained with haematoxylin and eosin. Scale bars = 200 μm. **e** Corpus luteum (CL) number measured in three representative sections of each ovary (*n* = 10 ovaries). **P < 0.01, ***P < 0.005 (hCG treated vs controls). **g** *Amh* expression levels in 2-month**-** and 6-month-old F1 and F2 ovaries, compared with their respective controls. *P < 0.05, **P < 0.01, ****P < 0.001

Then, we evaluated the fertility of 2-month-old F1 females and of 2- and 6-month-old F2 females (Fig. 3a) by mating them with control males (F1 or F2 controls) (*n* = 8, 10 and 19 for 2-month-old F1 control x F1 control, F1 x F1 control, and F2 x F2 control, respectively, and *n* = 6 and 7, for 6-month-old F2 control x F2 control and F2 x F2 control, respectively). All 2-month-old F1 and F2 females were mated within 1–2 weeks and showed the same full-term pregnancy frequency (100%) and comparable mean litter sizes (between 8 and 19 pups) as controls (Fig. 5a, b). On the other hand, the litter size of 6-month-old F2 females was significantly smaller than that of 6-month-old controls (fewer than ten pups for five of the seven females, 71%), whereas the mating time was not significantly different compared with controls (Fig. 5a, b). This suggests normal reproductive capacity in 2-month-old F1 and F2 females, but subfertility in 6-month-old F2 females.

Therefore, we collected ovaries from 2- and 6-month-old control, F1 and F2 mice. Haematoxylin-eosin (H&E) staining showed a similar histology of 2-month-old F1 (in utero exposed) and control ovaries, with similar numbers of growing follicles and CLs (Fig. 5c; Supplementary Fig. 4a–c), confirming normal sexual maturity in both groups. Conversely, 2-month-old F2 ovaries contained significant higher numbers of follicles and CLs (Fig. 5c; Supplementary Fig. 4a–c). Moreover, stimulation with human chorionic gonadotropin (hCG) to induce follicle growth and ovulation, increased the number of CLs in 2-month-old control and F1 ovaries, but not in 2-month-old F2 ovaries (Fig. 5c–e). In 6-month-old F2 ovaries, CL number also was strongly increased (Fig. 5f), although the number of follicles remained similar as in controls (Fig. 5f; Supplementary Fig. 4a–c). The percentage of atretic follicles (pre-antral or antral follicles) was significantly higher in 2- and 6-month-old F2 ovaries than in their respective controls (Supplementary Fig. 4d). Moreover, at both ages, expression of *Amh*, a marker of the follicle reserve^51^, was significantly lower in F1 and F2 ovaries than in their respective controls (Fig. 5g). This suggests that the enhanced PF formation and the impaired transition from PF to primary follicles in early postnatal ovaries progressively lead to a decrease of the ovarian reserve between 2- and 6-month of age. This accelerated ovarian aging^35^ might explain the subfertility of 6-month-old F2 females.

### Luteolysis is impaired in F2 females

**Fig. 6:**
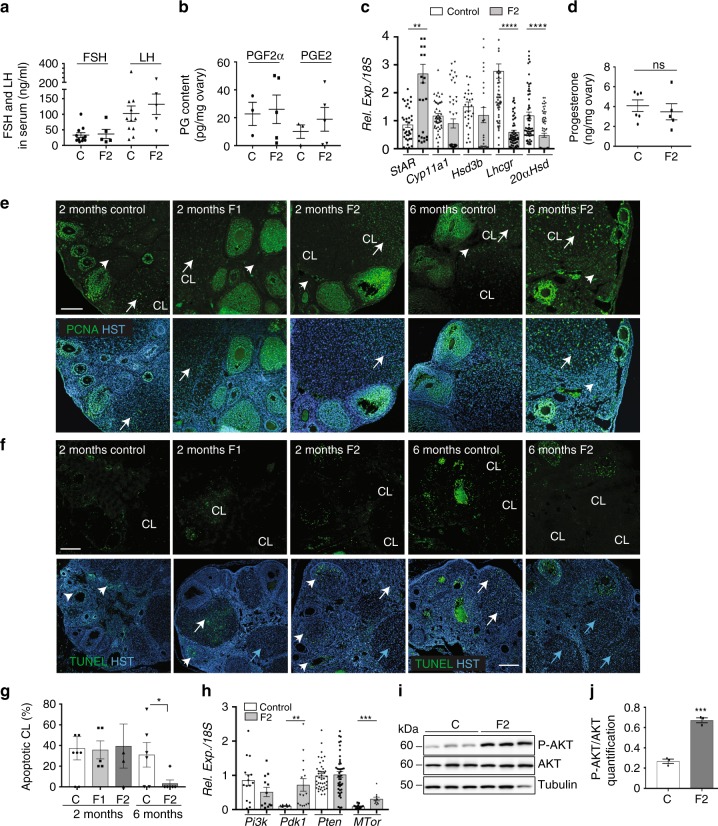
Luteolysis is impaired in 6-month-old F2 ovaries. **a** FSH and LH secretion in serum samples from 6-month-old ovaries measured by ELISA (*n* = 11 control (C) and *n* = 5 F2 ovaries). **b** PGF_2α_ and PGE_2_ production in 6-month-old ovaries measured by ELISA (*n* = 3 control (C) and *n* = 5 F2 ovaries). **c** Relative expression levels of the steroidogenic genes *StAR, Cyp11a1, Hsd3β, Lhcgr* and *20αHsd* in 6-month-old F2 ovaries normalised to *18S*; **P < 0.01, ****P < 0.001. **d** Progesterone secretion was measured by LC/MS in 6-month-old control (*n* = 6) and F2 (*n* = 5) ovaries; ns   not significant. **e** Proliferation in 2-month-old F1 and F2 and in 6-month-old F2 ovaries was assessed by immunofluorescence using anti-PCNA (green) antibody and Hoechst (HST, blue); CL = corpus luteum; white arrows highlight proliferating CLs and arrowheads indicated stromal cells. **f**, **g** Apoptotic cells in 2-month-old F1 and F2 and in 6-month-old F2 ovaries were visualised by TUNEL assay (green) and nuclear staining with Hoechst (HST, blue) (**f**) and apoptotic corpora lutea (CL) were counted (**g**); the data are presented as the percentage of apoptotic CLs. *P < 0.05 (**g**). White and blue arrows indicate apoptotic and non-apoptotic CLs, respectively, and arrowheads indicate growing follicles (**f**). Scale bars = 300 μm. **h**–**j** The AKT-PDK1 pathway is activated in 6-month-old F2 ovaries compared with controls. Relative expression levels of *Pi3k*, *Pdk1*, *Pten* and *Mtor* normalised to *18S*, in control and F2 ovaries; **P < 0.01, ***P < 0.005 (**h**). Representative immunoblots of total AKT, phosphorylated AKT (P-AKT) and tubulin (**i**) show that the AKT pathway is activated in F2 ovaries (*n* = 3), as indicated by the increased P-AKT/AKT ratio compared with control (*n* = 4); ***P < 0.005 (**j**)

In the 6-month-old F2 group, CL number was significantly higher than in controls (Fig. 5f; Supplementary Fig. 4c). This suggests that follicle recruitment was increased or that CLs from the previous follicular cycles did not regress. The level of FSH and LH secretion in the serum (Fig. 6a) was not significantly different between F2 and control animals, suggesting an intact regulated hypothalamic–pituitary axis. Normally, CL functional and structural regression is triggered by PGF_2α_ that inhibits LH-stimulated steroidogenesis and progesterone secretion and stimulates luteal cells apoptosis^56,57^. During human CL maturation and regression, the main PGs produced in luteal tissue changes from the luteotrophic PGE_2_ to the luteolytic PGF_2α_^58^. PGE_2_ and PGF_2α_ levels were comparable in F2 and control ovaries (Fig. 6b). Conversely, the gene encoding steroidogenic acute regulatory protein (*Star*) was significantly upregulated, whereas the genes encoding luteinizing hormone/chroriogonadotropin receptor (*Lhcgr*) and aldo-keto reductase family 1, member C18 or 20α-hydroxysteroid dehydrogenase (*20αHsd* or *Akr1c18),* the major progesterone degradation enzyme^30^, were strongly downregulated (Fig. 6c). Expression of the genes encoding cholesterol side-chain cleavage enzyme (*Cyp11a1*) and 3β-hydroxysteroid dehydrogenase (*Hsd3b*) was not modified (Fig. 6c). However, progesterone production was similar in 6-month-old control and F2 ovaries (Fig. 6d), suggesting that functional luteolysis was not impaired.

Histology analysis of new and old CL in F2 ovaries using the method described by Taketa et al.^59^ showed that 97% of CLs in F2 ovaries were old CLs (3% of new CLs), whereas in control ovaries, 51% of CLs were old and 49% were new CLs (Supplementary Fig. 4e). Analysis of CL proliferation/apoptosis using an antibody against proliferating cell nuclear antigen (PCNA) and the TUNEL assay showed that in 2-month-old ovaries, PCNA was localised in granulosa and theca cells of all follicle types in the control, F1 and F2 groups (Fig. 6e). Differentiated luteal cells were PCNA-positive in 50% of all CLs, and most CLs showed TUNEL-positive cells (Fig. 6e, f), suggesting that structural luteolysis proceeded normally in 2-month-old ovaries. In contrast, in 6-month-old F2 ovaries, we could not detect any TUNEL-positive cell (Fig. 6f, g), and 100% of luteal and stromal cells were PCNA-positive, differently from controls (Fig. 6e). As AKT-PDK1 pathway activation promotes cell proliferation and cell survival^60^, particularly in ovary^29,61^, we analysed its status in 6-month-old ovaries. We found that *Pdk1* and *Mtor* were significantly upregulated in 6-month-old F2 ovaries compared with control (Fig. 6h). Western blotting with anti-AKT and -phosphorylated AKT antibodies revealed that the AKT pathway was significantly activated in the F2 group compared with control (Fig. 6i, j; Supplementary Fig. 5), suggesting that, in addition to the lack of apoptosis, increased AKT-mediated cell survival also might contribute to inhibiting CL structural regression in 6-month-old F2 ovaries.

## Discussion

In this study, we demonstrated that APAP + IBU exposure during the developmental period of sex determination produces intergenerational effects on female reproductive development. We analysed for the first time the molecular mechanisms involved in these effects, highlighting that the in utero exposure to this drug combination modifies activation of the AKT signalling pathway in postnatal and adult ovaries. Upon in utero APAP + IBU exposure, germ cell proliferation and meiosis entry were affected in embryonic ovaries, leading to abnormal PF formation and activation in postnatal ovaries. Consequently, the ovarian reserve decreased in exposed (F1) adult animals and their offspring (F2), leading to subfertility of 6-month-old F2 animals that showed accelerated ovarian aging and abnormal CL persistence. These results raise the question of whether in utero exposure to APAP + IBU could harm the reproductive lifespan also in women. Nearly 4% of pregnant women use a combination of APAP and NSAIDs. This drug combination was associated with shorter anogenital distance in the exposed male offspring^2^. Also, APAP and IBU are amongst the drugs most frequently present in the natural environment, including surface and groundwater in many countries, and they are not eliminated during the wastewater treatment process^62,63^. This suggests an uncontrolled and continuous sub-therapeutic dose exposure of the general population, including pregnant women and young pre-pubertal individuals.

We found that exposure of mouse embryos to therapeutic doses of APAP or IBU alone did not affect embryonic germ cells, in contrast to others studies performed either using sub-toxic doses of APAP^15^ in rats or lower doses for longer time in mice^14^, or using ex vivo cultures of fetal human ovaries^16,17^. Conversely, exposure to the APAP + IBU combination during early embryogenesis led to increased proliferation of embryonic germ cells, delayed meiosis initiation and progression, and altered production of the PGs PGD_2_, PGE_2_ and PGI_2_. This suggests that these PGs play essential roles in fetal female germ cell development.

The APAP + IBU combination was administered at 10.5–13.5 dpc, when female PGCs have reached the genital ridge and still divide, and when DNA methylation was erased^64^ at meiosis initiation. The decreased expression of *Stra8* and of the pluripotency genes *Pouf5 (Oct4)* and *Dppa4* could disturb the onset of meiotic prophase I at 12.5 dpp that normally proceeds in female PGCs^23^. The increased cell proliferation and putative modifications of epigenetic marks induced by in utero APAP + IBU exposure might affect directly or indirectly germ cell development. Deposition of the histone mark H3K4me3 on meiotic gene promoters, which normally favours their expression and is essential for meiosis timing, could be disturbed^65^. For instance, *Stra8* expression can be influenced by changes in DNA methylation of its promoter upon exposure to various endocrine disruptors^66^. Additional studies are needed to investigate the epigenetic mechanisms modified by APAP–IBU exposure. Concomitantly, some genes involved in meiosis I prophase progression that were shown to be targets of STRA8^40,67^ were upregulated. Among these genes, *Meioc* encoding an RNA-binding protein might be involved in regulating meiotic cell cycle^68^ and meiotic recombination^69^ transcripts, via post-transcriptional mechanisms, even in presence of lower levels of *Stra8* expression. However, despite the variations in gene expression, oocytes can complete meiotic prophase in postnatal exposed ovaries.

As completion of the first meiotic prophase is tightly correlated with the initiation of follicular assembly^70^, oocytes arrested at the diplotene/dictyate stage are competent to direct follicle assembly in APAP + IBU-exposed ovaries. After birth, germline cysts breakdown into individual oocytes that become surrounded by somatic pre-granulosa cells to form PFs. During this process, only a third of the initial oocytes survives^24^. The increased number of germ cells in cysts in exposed 17.5 dpc ovaries led to an increase of the PF pool in 8dpp F1 (exposed) and also in F2 ovaries. In utero APAP–IBU exposure could disturb the regulation of the cell survival and/or programmed cell death pathways, leading to the loss of some oocytes during the first days after birth^24,25^, as well as to the expression of *Sohlh1* and *Figla* that activate PF formation^49,50^. These initial steps in PF formation are critical because they are needed to ensure the regular follicular development throughout the reproductive life^71^. Differently from our results, a previous study showed that exposure to APAP alone between 7 and 13.5 dpc induced a reduction of the PF number, leading to decreased fertility in 6 and 10-month-old mice^14^. Oocyte and PF activation prior to selection in the growing follicle pool^26^ also were enhanced upon in utero exposure to APAP + IBU. We also found that increased apoptosis in growing follicles and inhibition of the AKT signalling pathway (decreased phosphorylated AKT/AKT ratio) upon APAP + IBU exposure led to inhibition of FOXO3 nucleocytoplasmic translocation. Consequently, the number of primary and secondary follicles was reduced in the follicle pool. In oocytes, the PI3K/AKT/FOXO3 signalling cascade is the main pathway that regulates PF activation and survival^29^. Deletion of genes encoding PTEN^53^ or signalling molecules downstream of PI3K, such as PDK1^72^ or FOXO3A^73^, in mouse oocytes leads to abnormal PF development and POI in adult ovaries. The increased PF pool followed by higher apoptosis in postnatal F1 (directly exposed) and F2 ovaries suggests an intergenerational transmission of these effects.

Finally, we observed that the ovarian reserve decreased between 2 and 6 months of age and led to subfertility in 6-month-old F2 females. Ovarian aging or menopause occurs when the PF pool is exhausted, and abnormal PF development causes POI in adult ovaries^35^. The significant increase of CL number in 6-month-old F2 ovaries might be the consequence of the AKT pathway activation leading to increased luteal cell proliferation and survival^29,61^, rather than being due to a higher number of ovulations because FSH and LH secretion was normal. This, together with the lack of luteal cell apoptosis, led to the structural maintenance of CLs that should normally have regressed in the absence of pregnancy to allow the onset of a new cycle^30^. The maintenance of progesterone secretion in F2 could result from an alteration of CL function that was compensated by the increased number of CLs and the strong downregulation of the *20αHsd* gene, which encodes a protein to catabolize progesterone. Moreover, increased expression or activity of other enzymes involved in progesterone metabolism, such as 5α-reductases, 17α-hydroxylase or 3α-hydroxysteroid dehydrogenase^74,75^ in F2 ovaries, might explain why progesterone production was not increased in F2 ovaries compared with controls, although they contained a larger number of CLs. CL persistence was probably due to the inhibition of the activity of PGF_2α_, the major luteotrophic factor that was normally produced. Its inhibition could also promote AKT activation in F2 ovaries^76^. Moreover, progesterone, which is the main steroid produced by the CL^30^, plays an important role in stimulating its own secretion and in protecting the CL from programmed cell death^77^. Consequently, it may also contribute to CL maintenance in F2 ovaries.

In conclusion, we showed that in the mouse, exposure to the widely used APAP + IBU combination has an important impact on germ cell development in embryonic ovary, and on their maturation and folliculogenesis in postnatal and adult ovaries and can also induce inheritable effects. These effects lead to subfertility in 6-month-old F2 animals that show an accelerated ovarian aging. Additional studies are now required to identify the modifications of the primordial germ cell epigenome associated with such intergenerational effects, as shown after exposure to various endocrine disruptors^78^. As the global dynamics of the main epigenetic mechanisms are conserved between mouse and humans^79^, this mouse model of in utero NSAID/APAP exposure provides a valuable tool for investigating their effects, and also those of other drugs and environmental chemicals, on human female reproduction and fertility.

## Methods

### Animals and experimental protocols

For all experiments, wild-type pregnant CD1 females (5.5 dpc of pregnancy) were obtained from Janvier Laboratories (Janvier Labs, Le Genest-Saint-Isle, France) and maintained in the animal care facility for 5 days prior to treatment. Animals were kept and bred at the IGH animal care facility in controlled environmental conditions. All animal experiments were conducted according to procedures approved by the Réseau des Animaleries de Montpellier (RAM) (agreement number 34–366 for B.B.-B.) and by the Regional Ethics Committee. APAP and IBU stock solutions (500 and 219 mM, respectively) (MilliporeSigma, Burlington, MA, USA) were prepared in ethanol and 10-fold diluted with PBS before oral administration between 10.5 and 13.5 dpc. For experiments with single drugs, 30 mg/kg APAP, 15 mg/kg IBU^11,14^ or 9.7% ethanol (control) were administered daily twice (150 μl by gavage, 6 h apart) (*n* = 6 independent experiments with two pregnant females for each experiment and condition; thus, *n* = 12 F0 females in total per group). For experiments with two drugs [30 mg/kg/d APAP + 15 mg/kg/d IBU or ethanol as control], the same doses of each drug were alternately administered every 3 h (four times, 150 μl by gavage) (*n* = 4 independent experiments with two pregnant females for each experiment and condition; thus *n* = 8 F0 females in total per group). These experiments generated the F1 (exposed) embryos/pups and the F1 animals. To analyse the intergenerational effects of APAP + IBU exposure, F2 animals were generated by breeding 2-month-old F1 males and F1 females (*n* = 15 matings) whereas F2 controls were generated from F1 male and F1 female controls (*n* = 7 matings) (Fig. 3a); neither siblings nor cousin animals were crossed to avoid inbreeding artifacts.

Ovaries from 13.5 dpc-17.5 dpc F1 embryos, 8-14-21-30 dpp F1 and F2 pups and 2- and 6-month-old F1 and F2 mice were collected. For each embryo/pup/animal, one ovary was fixed in 4% paraformaldehyde (PFA)/phosphate-buffered saline (PBS) and processed for paraffin inclusion, whereas the second ovary was immediately frozen at −80 °C for RNA extraction. For the analysis, *n* = 10–16 F1 embryonic gonads from two gestated females and *n* = 4–10 postnatal gonads were pooled from two litters, for each independent experiment (*n* = 6 with single drugs and n = 4 with double drugs). F1 and F2 adult ovaries (*n* = 6 to 10) from different litters were processed independently.

EdU (5-ethynyl-2-deoxyuridine) (2.5 mM/150 μl) was intraperitoneally injected in F0 pregnant females 2 h before killing, and EdU-positive cells were detected using the Click-iT EdU Assay (Thermo Fisher Scientific, Waltham, MA, USA), according to the manufacturer’s instruction. Female mice (8 weeks) were treated with 10 IU of human chorionic gonadotropin (hCG) (Sigma-Aldrich), a gonadotropin with both FSH- and LH-like properties that promotes follicular growth, ovulation and luteogenesis^80^. Ovaries were dissected 48 h later for histological analysis.

### Histology, immunofluorescence, TUNEL and western blot analysis

Mesogonads from embryos, and postnatal and adult ovaries from F1 and F2 animals were collected, fixed in 4% PFA/PBS for 24 h, embedded in paraffin, sectioned (4 μm sections) and processed for histology or immunofluorescence. Sections were stained with H&E using standard protocols. Immunofluorescence was performed as previously described^11,81^ and detailed in Supplementary Table 3 with the information about the antibodies used.

Apoptosis in follicles and CLs was determined by TUNEL assay with the DeadEnd fluorometric TUNEL kit (Promega). Individual sections (*n* = 1 for follicles; *n* = 3 for CLs (40 μm apart)) of ovaries (*n* = 3 postnatal ovaries and *n* = 6 adult ovaries; *n* = 4 experiments) were analysed. Briefly, deparaffinated and rehydrated sections were incubated with proteinase K for 4 min, and fixed again with 4% PFA/PBS. Fragmented DNA of apoptotic cells was detected by incorporation of fluorescein-12-dUTP at the 3′-OH DNA ends using the terminal deoxynucleotidyl transferase, recombinant, enzyme (rTdT) at 37 °C for 1 h. After stopping the reaction by immersion in 2× SSC solution, sections were rinsed in PBS and stained with Hoescht (HST). The fluorescein-labelled DNA and HST staining were visualised by fluorescence microscopy; the results were expressed in percentage of TUNEL-positive follicles or CLs among total number of follicles or CLs per section.

Protein extracts from 8 dpp and 6-month-old ovaries were prepared and analysed by western blotting (SDS PAGE electrophoresis), as previously described^82^. Protein contents were quantified using the Image Lab software 5.1 (Biorad).

### Analysis of meiotic prophase by chromosome spreads

Meiotic spreads on oocytes from F1 13.5 dpc (preleptotene/leptotene oocytes) and F1 17.5 dpc (zygotene/pachytene) ovaries were prepared as described in ref. ^83^. Immunostaining was performed using a milk-based blocking buffer (5% milk, 5% donkey serum in 1 × PBS). Spreads were incubated with the guinea pig anti-SYCP3^84^, mouse monoclonal anti-phospho-H2AX (Upstate) and rabbit anti-SYCP1 primary antibodies (Supplementary Table 3) at room temperature overnight. Anti-guinea pig Alexa Fluor 488, anti-mouse Alexa Fluor 647 and anti-rabbit Alexa Fluor 555 secondary antibodies (Molecular Probes) were added at 37 °C for 1 h. Nuclei were stained with DAPI (4′-6-Diamidino-2-phenylindole, 2 μg/ml) during the final washing step.

Meiotic prophase I staging of mouse oocytes was performed using these SYCP3, SYCP1 and γH2AX markers^85,86^ and between 85 and 155 oocytes were analysed in each ovary (*n* = 9 for control and APAP + IBU conditions, from *n* = 3 litters). For F1 13.5 dpc oocytes, the preleptotene (appearance of heterochromatin patches in the nucleus), early/mid leptotene (small stretches of SYCP3 indicating onset of axial element formation and increase in the number and intensity of γH2AX-positive domains indicating DNA double-strand break formation) and late leptotene (fully formed axial elements stained by SYCP3 but lacking SYCP1, indicating absence of synapsis) stages were defined. For F1 17.5 dpc oocytes, four stages were defined: leptotene, early/mid zygotene (synapsis has started and short SYCP1 stained structures are visible), late zygotene (the meiosis-specific chromosome axes visualised by SYCP3 are at 50% associated in the synaptonemal complexes (SC) visualised by SYCP1) and pachytene (100% of the SCs are synapsed and are stained by SYCP3 and SYCP1, indicating full synapsis).

### Quantification of cells, cysts, oocytes, follicles and corpora lutea

In embryonic gonads, proliferative FOXL2 + granulosa cells and VASA + germ cells were quantified by counting proliferating EdU + cells amongst all FOXL2 + or all VASA + cells. For each data point with single drug, n = 20 to 25 F1 embryonic ovaries (from *n* = 12 litters) were analysed and for each data point with double drugs, *n* = 16 to 20 F1 embryonic ovaries (from *n* = 8 litters) were analysed (between 400 and 2100 cells were counted).

Meiosis prophase I establishment was analysed by immunofluorescence analysis of γH2AX, SYCP3 and DMRT1 expression, and meiotic cell number was evaluated by counting γH2AX-, SYCP3- and DMRT1-positive cells among all VASA + cells in F1 13.5 and 17.5 dpc gonads. For each data point, *n* = 16 to 24 gonads (400–900 germ cells) were analysed. The number of oocytes in cysts in F1 17.5 dpc embryonic gonads was determined on sections immunostained with anti-VASA and anti-laminin (that labelled the cyst basal membrane) antibodies. One representative section of control (*n* = 10) and exposed (*n* = 12) ovaries from three litters was analysed per data point (*n* = 3–5 cysts counted per section).

Follicles were classified as follows: primordial follicles (oocytes surrounded by a single layer of flat granulosa cells), primary follicles (oocytes surrounded by one layer of cuboidal granulosa cells) or secondary follicles (oocytes surrounded by more than one layer of granulosa cells), according to the criteria established by Pedersen and Peters^87^. This analysis was performed in a double-blind manner using the Nanozoomer Digital Pathology (NDPview2) software (Hamamatsu). Only oocytes with a visible nucleus were counted. Follicles and corpora lutea were counted on three H&E-stained sections for each ovary (sections separated by 20 μm or 40 μm for CLs count). At least six adult ovaries (*n* = 6 to 10 for F1 and F2 ovaries) were analysed and *n* = 16 to 20 F1 and F2 postnatal ovaries from different litters were analysed, per data point. The results were expressed as the percentage of primordial/primary/secondary follicles relative to the total number of follicles and per histological section.

Oocytes diameter in primordial follicles of 8 and 14 dpp postnatal ovaries (PFs were visualised by the presence of round oocytes surrounded by one layer of flat granulosa cells) was determined on H&E-stained sections using the NDPview2 software. Each data point was the analysis of 200 oocytes (from three sections separated by 20 μm of *n* = 20 different ovaries per condition).

Concomitantly, the different CL morphologies were evaluated on H&E-stained sections of 6-month-old control and F2 ovaries. In contrast with new corpora lutea (new CL) that display strong nuclear staining by HE, corpora lutea formed in previous cycles (old CL) showed cells with heterogeneous shapes and sizes with increasing cytoplasmic area^59^. The numbers of new CLs and old CLs per section were calculated by averaging their number in three sections of each ovary (*n* = 6 per data point).

TUNEL-positive follicles were counted on one section of postnatal ovaries (*n* = 12) for each condition. TUNEL-positive CLs were counted on three sections (40 μm apart) for each F1 and F2 ovary (*n* = 6 for each type). The data were presented as the percentage of apoptotic follicles/CLs relative to the total number of follicles/CLs per section.

### RNA isolation, RT-qPCR and RNA-seq analysis

For the gene expression analysis, *n* = 10–16 F1 embryonic gonads from two gestated females and *n* = 4–10 postnatal gonads were pooled from two litters, for each independent experiment (*n* = 4 with double drugs) to have four biological replicates. Specifically, embryonic and postnatal ovary RNA was extracted from the control and APAP + IBU-exposed pools (*n* = 4 control and *n* = 4 APAP + IBU). F1 and F2 adult ovaries were processed independently (*n* = 6–10 per condition from different litters). RNA was extracted from gonads using TRIZOL (Thermo Fisher Scientific) and processed as described previously^11^. RNA quality was controlled with the Agilent 2100 Bioanalyzer system. Real-time RT-qPCR was performed as previously described^81^ using the primers listed in Supplementary Table 4 and *Rps29* or *18S* as the reference gene for data normalisation in embryonic gonads^88^ and postnatal/adult samples, respectively.

For RNA-seq experiments, the total RNAs (three out of the four pools) from embryonic gonads (*n* = 3 control and *n* = 3 APAP + IBU) were independently processed to provide replicates. RNA-seq libraries were generated from 300 ng of the total RNA using the TruSeq Stranded mRNA LT Sample Preparation Kit (Illumina, San Diego, CA), according to the manufacturer’s instructions. Briefly, following purification with oligo-dT attached to magnetic beads, mRNA was fragmented using divalent cations at 94 °C for 2 min. Cleaved RNA fragments were copied into first-strand cDNA using reverse transcriptase and random primers. Strand specificity was achieved by replacing dTTP with dUTP during the second-strand cDNA synthesis using DNA Polymerase I and RNase H. Following the addition of a single ‘A’ base and subsequent ligation of the adapter on double-stranded cDNA fragments, products were purified and enriched by PCR (98 °C for 30 s; [98 °C for 10 s, 60 °C for 30 s, 72 °C for 30 s] × 12 cycles; 72 °C for 5 min) to create the cDNA libraries. Surplus PCR primers were further removed by purification using AMPure XP beads (Beckman-Coulter, Villepinte, France), and the final cDNA libraries were checked for quality and quantified using capillary electrophoresis (Agilent 2100 Bioanalyzer). Libraries were then sequenced on an Illumina HiSeq 4000 system using paired-end 2 × 50 bp following Illumina’s recommendations.

Image analysis and base calling were performed using RTA 2.7.3 and CASAVA 2.17.1.14. Reads were mapped onto the mm10 assembly of the mouse genome using Tophat v2.0.14^89^ and the bowtie v2.1.0 aligner. Gene expression was quantified using HTSeq v0.6.1^90^, and gene annotations were from Ensembl release 86. Differential gene expression analysis was analysed using R and DESeq2 v1.6.3 Bioconductor library^91^.

### PG, steroids and serum hormone measurements

PGD_2_, 15-desoxy 12, 14-PGJ_2_, PGE_2_, PGF_2α_, 6-keto-PGF_1α_ (PGI_2_) and thromboxane (TxA2) were quantified in 13.5 dpc control and APAP + IBU-exposed ovaries by LC-MS/MS. For this experiment, 200 control and 210 exposed ovaries were isolated from embryos of 15 controls and 15 APAP + IBU-treated pregnant females and then pooled (*n* = 1 pool). Tissues were processed, and PGs were identified and quantified as previously described^11^. PGE_2_ and PGF_2α_ in 6-month-old control and F2 ovaries were measured by ELISA according to the supplier’s protocol (Cayman).

The concentration of follicle-stimulating hormone (FSH) and luteinizing hormone (LH) in serum was measured with specific EIA ELISA kit (ElabScience Biotech, E-EL-M0511 and E-EL-M0057 respectively, CliniSciences France). The mean sensitivities of these assays are 0.96 ng/ml for mouse FSH and 0.28 ng/ml for mouse LH. Briefly, blood samples collected from 6-month-old control and F2 females were allowed to clot for 60 min, followed by centrifugation at 2000 *g* for 20 min; serum was frozen at −80 °C until the FSH and LH assays.

Progesterone was quantified in 6-month-old control (*n* = 7), and F2 (*n* = 5) ovaries by liquid chromatography coupled to tandem mass spectrometry (LC-MS/MS). Control and F2 ovaries were homogenised in 2 × 600 μl of diethyl-ether. Homogenates were centrifuged at 20,000 *g* for 10 min, and supernatants were evaporated. Evaporated extracts were resuspended in 100 µl of water/acetonitrile (1/1, v/v) with 0.1% ammonia. Five microliters of D9-progesterone at 3 µg/mL were added. The sample extracts were then centrifuged at 20,000 *g* at 4 °C for 10 min. Finally, supernatants were transferred into new vials for analysis. Progesterone was quantified using D9-progesterone as internal standard and a linear regression with 1/X^2^ weighing in the range from 0.0053 to 6.395 ng. Five microliters extract was injected into the LC-MS/MS system that consist of a Waters ACQUITY UPLC^®^ System with an Acquity UPLC BEH C18 1.7 µm, 2.1 × 50 mm column and a reversed-phase gradient over a run time of 5 min. Initial conditions consisted of mobile phase A (0.1% ammonia in water) and mobile phase B (acetonitrile) with a column temperature of 50 °C and a flow rate of 0.600 mL/min. The gradient conditions ramped from 30% B to 100% B between 0.5 and 3 min, and were maintained up to 3.5 min, ramped to 30% and maintained up to 5.0 min for re-equilibration. The MS analysis was performed on a Waters XEVO™ TQ-S Mass Spectrometer operating in positive ion electrospray MRM mode. The monitored MRM transition was *m/z* 315.1 > 110 and 324.019 > 113.98, for progesterone and D9-progesterone, respectively. In these conditions, the mean retention time was around 2.08 min for the two compounds.

### Statistics and reproducibility

Statistical analysis was performed using GraphPad Prism 7. The Student’s *t* test was used to compare two groups in independent experiments and the one-way ANOVA test with the Tukey’s post hoc test for multiple comparisons (real-time quantitative RT-qPCR experiments and cell counting). A *P*-value < 0.05 was considered significant. For quantification of cells, follicles and oocytes, embryonic (*n* = 9–24) and postnatal (*n* = 16–20) ovaries from different litters were analysed whereas adult animals (*n* = 6–10 from different litters) were independently processed for each data point. Values represent the mean ± SEMs of these different ovaries. For gene expression studies, all values represent the mean ± SEM of *n* = 4 biological replicates and of three or more independent RT-qPCR experiments.

### Reporting summary

Further information on research design is available in the Nature Research Reporting Summary linked to this article.

## Supplementary information

Supplementary Information

Description of Additional Supplementary Files

Supplementary Data 1

Reporting Summary

## Data Availability

RNA-sequencing data as presented in Supplementary Data 1 and Supplementary Tables 1–3 are available at NCBI’s gene expression omnibus (GEO, http://www.ncbi.nlm.nih.gov/geo/query/acc.cgi?acc=GSE122547) with identifier GSE122547. All other relevant data generated or analysed during this study are included in this article and Supplementary Information are available from the corresponding author upon reasonable request.
