# Supplementary material for: In utero exposure to acetaminophen and ibuprofen leads to intergenerational accelerated reproductive aging in female mice
Source: Commun Biol. 2019 Aug 13;2:310. doi: 10.1038/s42003-019-0552-x (PMC6692356; doi:10.1038/s42003-019-0552-x)
Supplement: Supplementary file 1 — Supplementary Information [file 42003_2019_552_MOESM1_ESM.pdf]

Supplementary Information

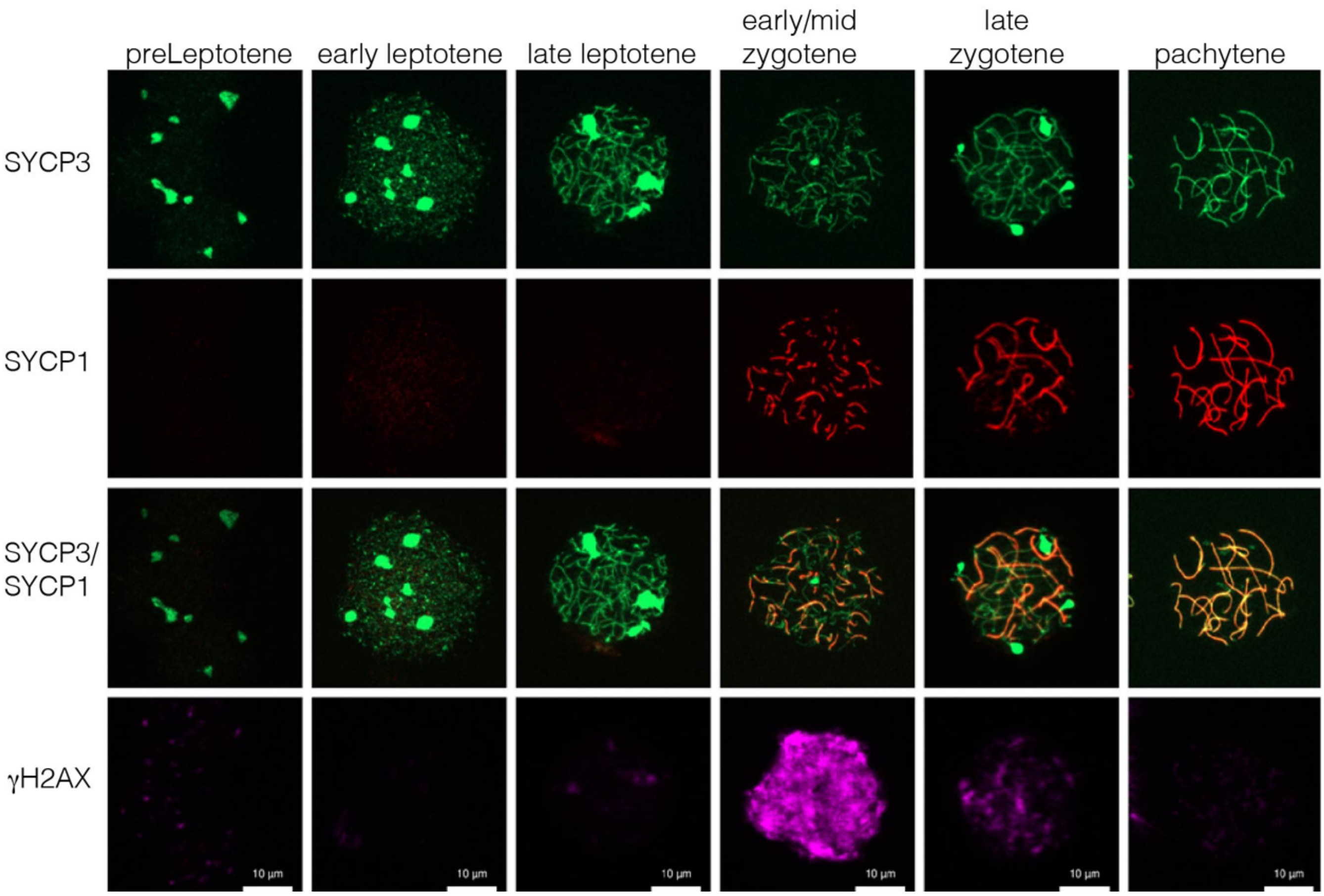

Supplementary Fig. 1. SYCP3, SYCP1 and γH2AX staining changes during meiotic prophase I substages.

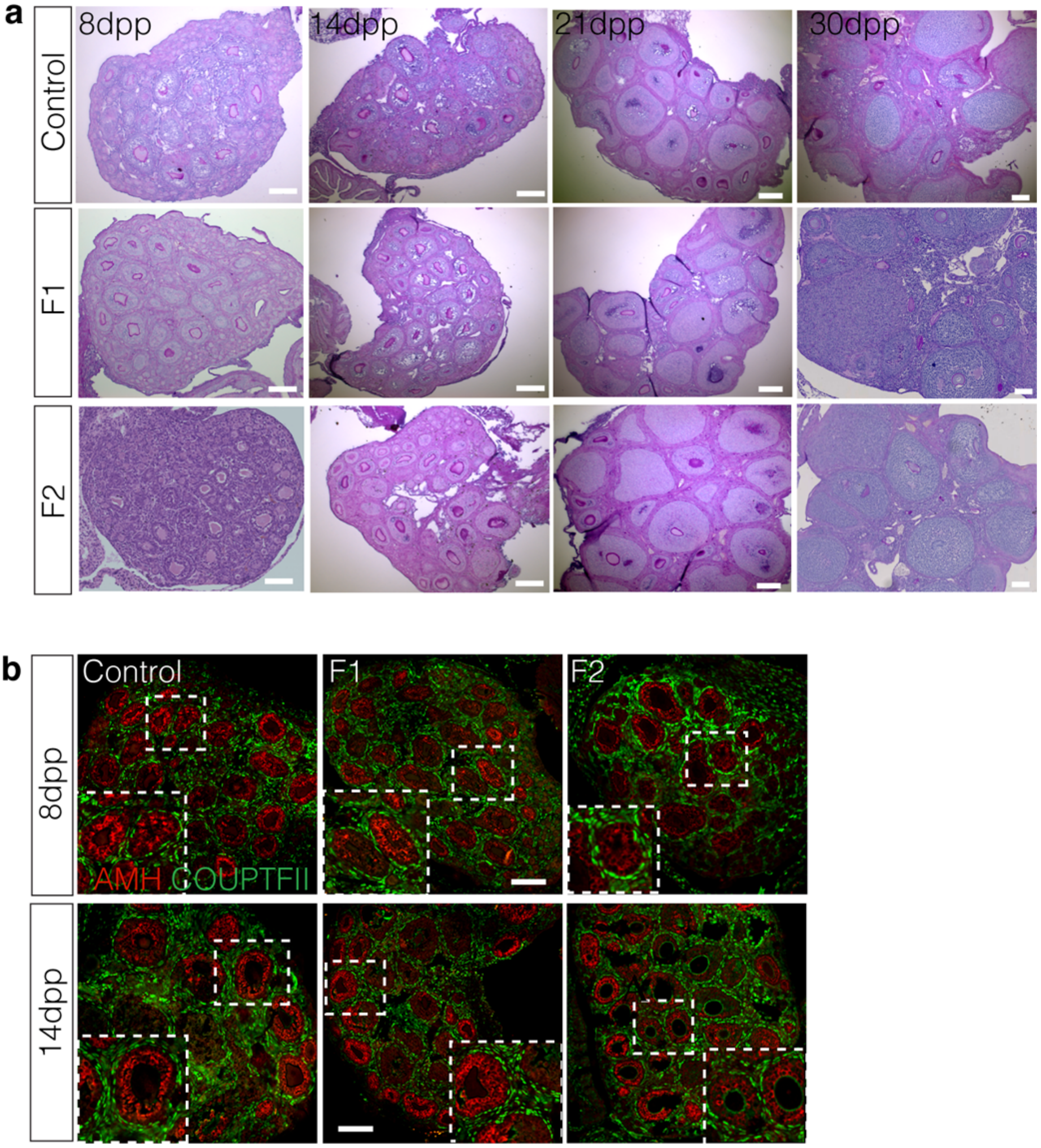

Supplementary Fig. 2. Follicular development in postnatal ovaries of *in utero* exposed animals and their offspring. **a** Representative images of paraffin-embedded tissue sections from 8, 14, 21 and 30dpp control, F1 and F2 ovaries, stained with the Periodic acid-Shiff dye. Scale bars= 80μm. **b** Representative immunofluorescence images for AMH (granulosa cell marker, red) and COUPTFII (interstitial cell marker, green) in tissue sections from 8 and 14dpp control, F1 and F2 ovaries. Enlarged panels show representative growing follicles. Scale bars=80μm.

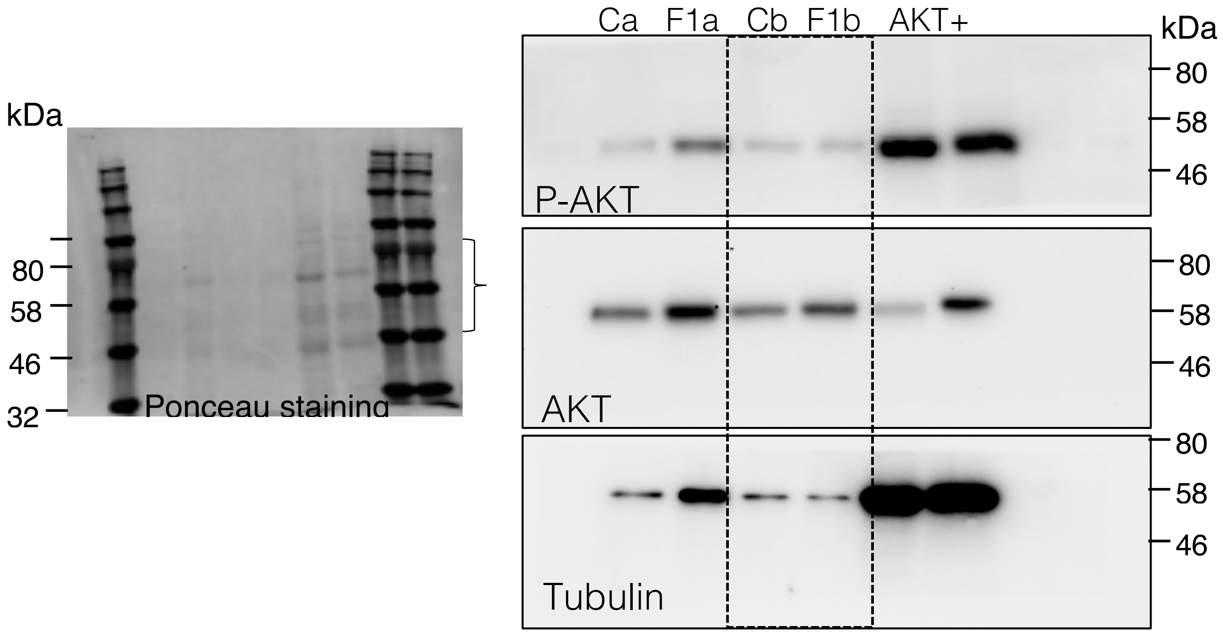

Supplementary Fig. 3. Full scans of images of Western blot data; C=8jpp control gonads, F1=8dpp F1 gonads; AKT+ is a positive AKT cell extract.

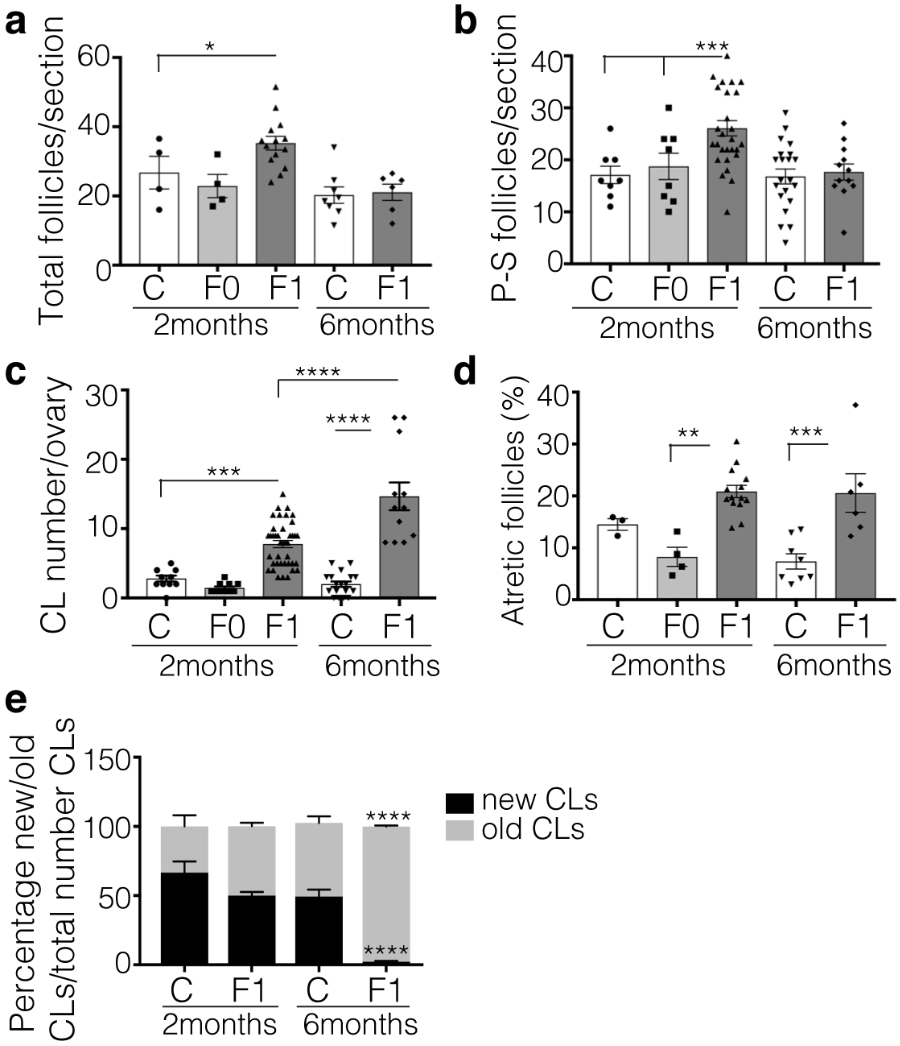

Supplementary Fig. 4. Follicle and corpus luteum (CL) counts in 2-month- and 6-month-old ovaries. **a** Total follicle number per section. **b** Primary (P) and secondary (S) follicle number per section. **c** CL number per section. **d** Percentage of atretic follicles relative to the total number of follicles. **e** Percentage of newly formed CLs (new CL) and CLs formed in previous cycles (old CL) in 2-month- and 6-month-old control and F2 ovaries. **a-e** Data are the mean ± SEM of all follicles and CLs on 3 sections from ovaries (n=6 to 10 animals per group), and *P <0.05, **P <0.01, ***P <0.005, ****P <0.001.

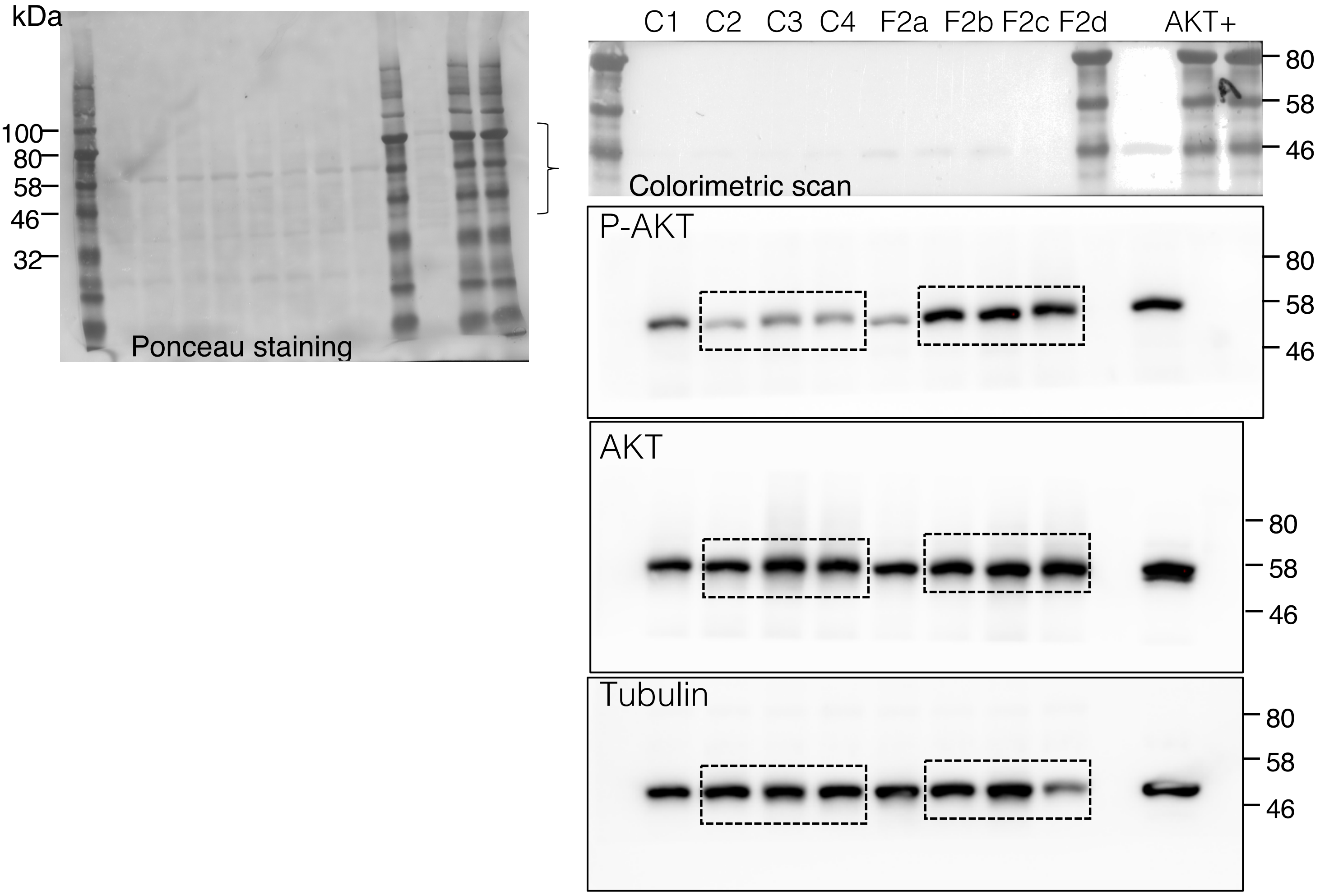

Supplementary Fig. 5. Full scans of images of Western blot data; C1-C4 and F2a-F2d are 6month-old control and F2 ovaries; AKT+ is a positive AKT cell extract.

Supplementary Table 1. Gene ontology analysis of differentially expressed genes in 13.5dpc APAP+IBU exposed ovaries compared with controls.

**Down-regulated genes/ GO biological process**	**Number of genes**
ribosomal small subunit assembly (GO:0000028)	19
ATP synthesis coupled proton transport (GO:0015986)	21
energy coupled proton transport, down electrochemical gradient (GO:0015985)	21
GTP biosynthetic process (GO:0006183)	10
deoxyribonucleoside monophosphate metabolic process (GO:0009162)	9
protein import into mitochondrial matrix (GO:0030150)	17
positive regulation of protein homooligomerization (GO:0032464)	11
mitochondrial electron transport, ubiquinol to cytochrome c (GO:0006122)	13
guanosine-containing compound biosynthetic process (GO:1901070)	13
cytoplasmic translation (GO:0002181)	62
ribosome assembly (GO:0042255)	70
NADH dehydrogenase complex assembly (GO:0010257)	42
mitochondrial respiratory chain complex I assembly (GO:0032981)	42
purine ribonucleoside biosynthetic process (GO:0046129)	20
cellular detoxification (GO:1990748)	20
purine nucleoside biosynthetic process (GO:0042451)	20
proteasomal ubiquitin-independent protein catabolic process (GO:0010499)	24
cellular response to superoxide (GO:0071451)	13
cellular response to oxygen radical (GO:0071450)	13
pyrimidine deoxyribonucleotide metabolic process (GO:0009219)	13
positive regulation of mitochondrial membrane potential (GO:0010918)	13
purine ribonucleoside triphosphate biosynthetic process (GO:0009206)	83
regulation of ubiquitin protein ligase activity (GO:1904666)	17

**Up-regulated genes/ GO biological process**	**Number of genes**
chromosome organization involved in meiotic cell cycle (GO:0070192)	71
homologous chromosome segregation (GO:0045143)	61
synaptonemal complex organization (GO:0070193)	25
meiotic chromosome segregation (GO:0045132)	93
male meiosis I (GO:0007141)	22
positive regulation of neuron migration (GO:2001224)	20
microtubule nucleation (GO:0007020)	20
reciprocal meiotic recombination (GO:0007131)	45
homologous recombination (GO:0035825)	45
meiosis II (GO:0007135)	12
negative regulation of translation, ncRNA-mediated (GO:0040033)	12
meiosis II cell cycle process (GO:0061983)	12
miRNA mediated inhibition of translation (GO:0035278)	12
centriole-centriole cohesion (GO:0010457)	12
regulation of translation, ncRNA-mediated (GO:0045974)	12
meiotic chromosome separation (GO:0051307)	23
synaptonemal complex assembly (GO:0007130)	22
DNA synthesis involved in DNA repair (GO:0000731)	28
resolution of meiotic recombination intermediates (GO:0000712)	19
synapsis (GO:0007129)	50
microtubule anchoring (GO:0034453)	25
miRNA metabolic process (GO:0010586)	16

Supplementary Table 2. Meiotic genes that are differentially expressed in 13.5dpc female gonads upon APAP+IBU exposure.

	**APAP+IBU downregulated genes**	**Fold change**
**Stra8**	stimulated by retinoic acid gene 8	0.76
**Rec8**	REC8 meiotic recombination protein	0.65

	**APAP+IBU upregulated genes**	**Fold change**
**Rad51ap2**	RAD51 associated protein 2	3.15
**Tex16**	testis expressed gene 16	2.92
**Sycp1**	synaptonemal complex protein 1	2.82
**Spata22**	spermatogenesis associated 22	2.66
**Hfm1**	HFM1, ATP-dependent DNA helicase homolog	2.47
**Sycp2**	synaptonemal complex protein 2	2.45
**Pet2**	plasmacytoma expressed transcript 2	2.43
**Rad21l**	RAD21-like (S. pombe)	2.41
**Fbxo47**	F-box protein 47	2.40
**Ccnb3**	cyclin B3	2.29
**Ugt8a**	UDP galactosyltransferase 8A	2.21
**Ccdc73**	coiled-coil domain containing 73	2.11
**Pramel1**	preferentially expressed antigen in melanoma-like 1	2.08
**Spdya**	speedy/RINGO cell cycle regulator family, member A	2.02
**Prdm9**	PR domain containing 9	2.01
**Msh4**	mutS homolog 4	1.98
**Tktl1**	transketolase-like 1	1.94
**Cdkl2**	cyclin-dependent kinase-like 2 (CDC2-related kinase)	1.91
**Meioc**	meiosis specific with coiled-coil domain	1.89
**D1Pas1**	DNA segment, Chr 1, Pasteur Institute 1	1.88
**Msh5**	mutS homolog 5	1.87
**Tsga10**	testis specific 10	1.80
**Usp32**	ubiquitin specific peptidase 32	1.79
**Sycp3**	synaptonemal complex protein 3	1.78
**Spo11**	SPO11 meiotic protein covalently bound to DSB	1.77
**Dmc1**	DNA meiotic recombinase 1	1.77
**Ddb2**	damage specific DNA binding protein 2	1.77
**Hormad1**	HORMA domain containing 1	1.74
**Dennd4a**	DENN/MADD domain containing 4A	1.72
**Dopey1**	dopey family member 1	1.72
**Caprin2**	caprin family member 2	1.71
**Adarb1**	adenosine deaminase, RNA-specific, B1	1.67
**Poln**	DNA polymerase N	1.67
**Taf7l**	TATA-box binding protein associated factor 7 like	1.62
**Cyld**	CYLD lysine 63 deubiquitinase	1,62
**Setdb2**	SET domain, bifurcated 2	1.57
**Inca1**	inhibitor of CDK, cyclin A1 interacting protein 1	1.55
**Crebl2**	cAMP responsive element binding protein-like 2	1.50
**Tex11**	testis expressed gene 11	1.49
**Tex12**	testis expressed gene 12	1.48
**Zfp541**	zinc finger protein 541	1.46
**Hormad2**	HORMA domain containing 2	1.45
**Dmrtc2**	doublesex and mab-3 related transcription factor like family C2	1.41
**Taf9b**	TATA-box binding protein associated factor 9B	1.40
**Ccdc36**	coiled-coil domain containing 36	1.37
**Stag3**	stromal antigen 3	1.37
**Madd**	MAP-kinase activating death domain	1.36
**Madd**	MAP-kinase activating death domain	1.36
**Syn2**	synapsin II	1.35

Supplementary Table 3. List of antibodies used for immunofluorescence (IF) experiments on tissues, western blotting (WB) and IF on chromosome spreads (SP).

Antigen	Species	Provider	Reference	Dilution WB/IF/SP
**FOXL2**	Rabbit	Gift from Dagmar Wilhelm	[1]	1/300
**AMH**	Rabbit	Gift from N. diClemente	[2]	1/400
**VASA/MVH**	Rabbit	Millipore	AB4330	1/300
**VASA/MVH**	Mouse	Abcam	ab27591	1/2000
**PCNA**	Mouse	Sigma-Aldrich	P8825	1/500
**FOXO3 (D19A7)**	Rabbit	Cell Signaling	12829	1/1000 WB<br>1/500 IF
**COUPTFII (NR2F2)**	Mouse	R&D Systems	T9026	1/300
**AKT(pan) (C67E7)**	Rabbit	Cell Signaling	4691	1/1000 WB
**Phosphorylated-AKT (Ser473)**	Rabbit	Cell Signaling	9271	1/1000 WB
**SYCP3**	Rabbit	Abcam	ab15093	1/200 (IF)
**SYCP3**	Guinea pig	Homemade	[3]	1/1000 (SP)
**SYCP1**	Rabbit	Abcam	ab15090	1/400 (SP)
**γH2AX**	Mouse	Sigma-Aldrich	05-636	1/1000 (IF)<br>1/10000 (SP)

Supplementary Table 4. Oligonucleotides used for qPCR. For each gene, the upper oligonucleotide corresponds to the forward primer and the lower to the reverse primer.

Gene	Sequence 5'-3'	Gene	Sequence 5'-3'
Rps29	tgaaggcaagatgggtcac<br>gcacatgttcagcccgtatt	*Akr1c18 (20αHsd)*	tggccctagccaagagttt<br>gccaattggaaatcaaagacc
18S	gatccattggagggcaagtct<br>ccaagatccaactacgagcttt	*ProgR*	tgcacctgatctaatcctaaatga<br>ggtaaggcacagcgagtagaa
Amh	ggggagactggagaacagc<br>agagctcgggctcccata	*StaR*	ttgggcatactcaacaacca<br>acttcgtccccgttctcc
FoxL2	cggggttcctcaacaactc<br>catctggcaggaggcgta	*Cyp11a1*	aagtatggccccatttacagg<br>tggggtccacgatgtaaact
Wnt4	gcgtagccttctcacagtcc<br>cgcatgtgtgtcaagatgg	*Lhcgr*	gatgcacagtggcaccttc<br>cctgcaatttggtggaagag
Rspo1	cgacatgaacaaatgcatca<br>ctcctgacacttggtgcaga	*Hsd3β*	gaccagaaaccaaggaggaa<br>gcactgggcatccagaat
FoxO1	cttcaaggataagggcgaca<br>gacagattgtggcgaattga	*Pten*	aggcacaagaggccctagat<br>ctgactgggaattgtgactcc
Pou5fl (Oct4)	cctgggggctctatttgg<br>ctcctgaagattttcattgttgtc	*Akt1*	tcgtgtggcaggatgtgtat<br>acctggtgtcagtctcagagg
Ddx4 (Vasa)	cgccaaacccttatgttcag<br>aaaaactctgcagccaacctt	*Akt2*	ataccaggcaccccttcct<br>cacaaagcataggcggtca
Dppa4	gctttcccagaacaaatgct<br>ttgtgagctgtcttcaacctg	*PI3K*	ccagacagtgtttttgtaagagga<br>tccatgccctatgcgact
Kitlg	tcaacattaggtcccgagaaa<br>actgctactgctgtcattcctaag	*Pdk1*	tgatgaagactgctatggcaac<br>cgtagacagggagtgggaag
Nobox	acggagaagctctgcaagaa<br>ctgtctccagtgcagcagtc	*MTor*	agaagacagcggggaagg<br>gcatcttgccctgaggttc
Figla	ggaagaagcgaaggtctcag<br>gtcagagggtctgccactgt	*FoxO3*	gctaagcaggcctcatctca<br>ttccgtcagtttgagggtct
Sohlh1	gagcgcgttgtcattcagt<br>ctggctgccatgagtgag	*Lhx8*	gatgggacgatgctgactg<br>ggtcattggatggggtaaca
Bmp15	acacagtaaggcctcccaga<br>tgctacctggtttgatgctaga	*Gdf9*	acccagcaaccaggtgac<br>cgatttgagcaagtgttccat

Supplementary References

1 Wilhelm, D. *et al*. Antagonism of the testis- and ovary-determining pathways during ovotestis development in mice. *Mech Dev* **126**, 324-336, doi:10.1016/j.mod.2009.02.006 (2009).

2 Tran, D., Picard, J. Y., Campargue, J. & Josso, N. Immunocytochemical detection of anti-mullerian hormone in Sertoli cells of various mammalian species including human. *J Histochem Cytochem* **35**, 733-743, doi:10.1177/35.7.3295030 (1987).

3 Grey, C., Baudat, F. & de Massy, B. Genome-wide control of the distribution of meiotic recombination. *PLoS Biol* **7**, e35, doi:10.1371/journal.pbio.1000035 (2009).
