# Supplementary material for: In utero exposure to acetaminophen and ibuprofen leads to intergenerational accelerated reproductive aging in female mice
Source: Commun Biol. 2019 Aug 13;2:310. doi: 10.1038/s42003-019-0552-x (PMC6692356; doi:10.1038/s42003-019-0552-x)
Supplement: Supplementary file 2 — Description of Additional Supplementary Files [file 42003_2019_552_MOESM2_ESM.docx]

**Description of Additional Supplementary Files**

**File Name** : Supplementary Data 1

**Description** : Up-regulated and down-regulated genes in APAP+IBU ovaries (p-value<5x10^-5^).
