# Supplementary material for: In utero exposure to acetaminophen and ibuprofen leads to intergenerational accelerated reproductive aging in female mice
Source: Commun Biol. 2019 Aug 13;2:310. doi: 10.1038/s42003-019-0552-x (PMC6692356; doi:10.1038/s42003-019-0552-x)
Supplement: Supplementary file 4 — Reporting Summary [file 42003_2019_552_MOESM4_ESM.pdf]

nature research

Corresponding author(s): Brigitte Boizet-Bonhoure

Last updated by author(s): Jul 15, 2019

Reporting Summary

Nature Research wishes to improve the reproducibility of the work that we publish. This form provides structure for consistency and transparency in reporting. For further information on Nature Research policies, see Authors & Referees and the Editorial Policy Checklist.

Statistics

For all statistical analyses, confirm that the following items are present in the figure legend, table legend, main text, or Methods section.

n/a	Confirmed	
☐	☒	The exact sample size (*n*) for each experimental group/condition, given as a discrete number and unit of measurement
☐	☒	A statement on whether measurements were taken from distinct samples or whether the same sample was measured repeatedly
☐	☒	The statistical test(s) used AND whether they are one- or two-sided<br>*Only common tests should be described solely by name; describe more complex techniques in the Methods section.*
☒	☐	A description of all covariates tested
☒	☐	A description of any assumptions or corrections, such as tests of normality and adjustment for multiple comparisons
☐	☒	A full description of the statistical parameters including central tendency (e.g. means) or other basic estimates (e.g. regression coefficient) AND variation (e.g. standard deviation) or associated estimates of uncertainty (e.g. confidence intervals)
☒	☐	For null hypothesis testing, the test statistic (e.g. *F*, *t*, *r*) with confidence intervals, effect sizes, degrees of freedom and *P* value noted<br>*Give P values as exact values whenever suitable.*
☒	☐	For Bayesian analysis, information on the choice of priors and Markov chain Monte Carlo settings
☒	☐	For hierarchical and complex designs, identification of the appropriate level for tests and full reporting of outcomes
☒	☐	Estimates of effect sizes (e.g. Cohen's *d*, Pearson's *r*), indicating how they were calculated

Our web collection on statistics for biologists contains articles on many of the points above.

Software and code

Policy information about availability of computer code

Data collection	The following software programs were used: Image J, Adobe Photophop CS5.1 and Exel. No custom made codes were used in this manuscript.
Data analysis	Adobe Photoshop was used. Statistical analysis was performed using GraphPad Prism 7. No custom made codes were used in this study.

For manuscripts utilizing custom algorithms or software that are central to the research but not yet described in published literature, software must be made available to editors/reviewers. We strongly encourage code deposition in a community repository (e.g. GitHub). See the Nature Research guidelines for submitting code & software for further information.

Data

Policy information about availability of data

All manuscripts must include a data availability statement. This statement should provide the following information, where applicable:

- Accession codes, unique identifiers, or web links for publicly available datasets
- A list of figures that have associated raw data
- A description of any restrictions on data availability

All relevant data generated or analyzed during this study are included in this article and Supplementary Information. All other data generated during this study are available from the corresponding author upon reasonable request. RNA-sequencing data as presented in Supplementary Data 1 and Supplementary Tables 1-3 are available at NCBI's gene expression omnibus (GEO, http://www.ncbi.nlm.nih.gov/geo/query/acc.cgi?acc=GSE122547) with identifier GSE122547.

Field-specific reporting

Please select the one below that is the best fit for your research. If you are not sure, read the appropriate sections before making your selection.

☒ Life sciences ☐ Behavioural & social sciences ☐ Ecological, evolutionary & environmental sciences

For a reference copy of the document with all sections, see nature.com/documents/nr-reporting-summary-flat.pdf

Life sciences study design

All studies must disclose on these points even when the disclosure is negative.

Sample size	For per os administration of APAP and IBU alone: n=6 protocols with two females/each condition, or APAP and IBU in combination: n=4 protocols (two females/each condition; same number of animals for control experiments.
Data exclusions	No data were excluded from the analyses.
Replication	Reproductibility of the experimental findings was found in all the performed protocols.
Randomization	Allocation of samples into experimental groups was random.
Blinding	The group allocation during data collection and analysis has been made in a blinded manner.

Reporting for specific materials, systems and methods

We require information from authors about some types of materials, experimental systems and methods used in many studies. Here, indicate whether each material, system or method listed is relevant to your study. If you are not sure if a list item applies to your research, read the appropriate section before selecting a response.

Materials & experimental systems

n/a	Involved in the study	
☐	☒	Antibodies
☒	☐	Eukaryotic cell lines
☒	☐	Palaeontology
☐	☒	Animals and other organisms
☒	☐	Human research participants
☒	☐	Clinical data

Methods

n/a	Involved in the study	
☒	☐	ChIP-seq
☒	☐	Flow cytometry
☒	☐	MRI-based neuroimaging

Antibodies

Antibodies used	All antibodies used in this study are described in Supplementary Table 3
Validation	All commercial antibodies have been validated by the manufacturer; both FOXL2 and AMH antibodies have been validated in the relevant citations as indicated in Supplementary Table 3.

Animals and other organisms

Policy information about studies involving animals; ARRIVE guidelines recommended for reporting animal research

Laboratory animals	Pregnant CD1 females were purchased from Janvier Laboratories
Wild animals	N/A
Field-collected samples	Animals were kept and bred at the IGH Animal Care Facility in controlled environmental conditions (Temperature 22°C, photoperiod 12/12h). Neither sibling nor cousin animals were crossed (F1 generation) to avoid inbreeding artifacts. Animals were humanely sacrified (isoflurane followed by cervical dislocation) before the removal of ovaries
Ethics oversight	All animal experiments were conducted according to procedures approved by the Réseau des Animaleries de Montpellier (RAM) (agreement number 34-366 for B.B.-B.) and by the Regional Ethics Committee.

Note that full information on the approval of the study protocol must also be provided in the manuscript.
